# A Case of Unilateral Pyonephrosis of Hematogenous Origin Treated by Nephrotomy

**Published:** 1913-11

**Authors:** R. M. Harbin

**Affiliations:** Rome, Ga.


### A CASE OF UNILATERAL PYONEPHROSIS OF HEMATOGENOUS ORIGIN TREATED BY NEPHROTOMY.

By R. M. Harbin, M. D.,
Rome, Ga.

Mrs. Blank, aged 34, was referred to me by her attending physicians, Drs. McLain and Carr, of Calhoun, Ga., and gave the following history. She has had four children, the youngest being five years old, and her previous history did not show anything except ill defined pelvic trouble. There had been slight bladder symptoms but no objective urinary deposits. On July 11th, 1913, she was taken with typhoid fever, which ran a typical course for twenty-six days. After a short period of irregular fever, her temperature remained normal for three or four days. On August 23rd she had a chill, followed by a temperature of 102; and these chills were repeated daily until September 1st, when she was admitted to the hospital with a temperature of 104 and pulse 136. Her facies indicated emaciation, sepsis, and she complained of pain in the region of the right kidney. The mass of the kidney was swollen, doughy and slightly movable, showing tenderness on pressure. There were neither objective nor subjective symptoms referable to the left kidney.

Urinalysis showed a Sp. Gr. 1008 and a trace of albumen with a few red blood cells, but no other deposits. A clinical diagnosis of pyonephrosis was made, and no further examination was attempted as the serious condition of the patient warranted dealing with her as an emergency case, and operation was promptly done.

A Robson incision was made on right loin, dissecting—carefully down to the right kidney. Adhesions were firm and numerous, especially at the upper pole, and in due time the kidney was delivered from its bed. The capsule showed one necrotic spot about 1½ inches in diameter near the upper pole, and was stripped off, revealing a similar condition of the kidney substance. There were three other smaller and similar lesions, and two spots of ecchymosis. The entire capsule was stripped off, and then Brodel's incision was made into the pelvis of the kidney, which was explored with the finger, but nothing was discovered. Nephrectomy did not seem advisable, and wound in the kidney and surrounding tissues were packed with gauze.

That operation lasted nearly a half hour, and there was no perceptible shock. The next day her highest temperature was 102 4-5, and lowest 98 4-5, and pulse 90 to 120. After an irregular course of fever, her temperature remained normal at the end of the first week. The second day after operation the blood count was as follows: Hemaglobin, 60%; white blood corpuscles, 10,400; red corpuscles, 3,715,000; polynuclear, 84.8%; and five days later blood analysis was about the same except that there were 13,500 white corpuscles. During the first twenty-four hours there was excreted 9.7 grams of urea, urine showing a small amount of albumen and pus cells with a few granular and hyaline casts. The second day gave 11.3 grams of urea, with a few hyaline and granular casts. The third day showed an excretion of 7.6 grams of urea, with less casts, and the fourth day 9.6 grams of urea. Urotropin was administered all along, and the quantity of excreted urine was now altogether satisfactory. The wound was packed every day, and showed leakage of urine, but healed in due time.

The condition of the urine at the time of operation, and the prompt subsidence of the symptoms of sepsis after nephrotomy and drainage warranted the belief that the left kidney was not involved to any appreciable extent, and a review of the case shows that the diagnosis of pyonephrosis may be made clinically and demonstrates the value of prompt surgical interference.

A report from her two months later states that she is entirely well.

